# Space tourism flow generated from social media data

**DOI:** 10.1016/j.dib.2023.109061

**Published:** 2023-03-15

**Authors:** Kang-Lin Peng, Xianglin Chan, Chih-Hung Wu

**Affiliations:** a Faculty of International Tourism and Management, City University of Macau. Avenida Padre Tomás Pereira Taipa, Macau SAR, China; b Department of Digital Content and Technology, National Taichung University of Education, Taiwan

**Keywords:** Space tourism, Behavioral intention, Flow, Social media, Attitude

## Abstract

The data is for empirical studies derived from a related research article about space tourism [1], which is a conceptual article with a different aim of economic measurement scale. Most space tourism research is conceptual because data from the nascent industry is limited [2]. Thus this data is constrictive for conducting empirical studies to contribute to quantitative analysis in space tourism [3]. Data from this study were collected by recruiting 361 respondents through snowball and convenient sampling targeted to samples interested in space tourism; 339 responses were adopted after the valid screening of missing data or data bias [4]. Targeted groups of potential space tourism customers were investigated to collect data through a designed questionnaire on the platform, Wenjuanxing, with a majority population database providing functions equivalent to Amazon Mechanical Turk [2]. The reliability and validity of all constructs showed that the questionnaire was proper for measurement [3]. Data analysis applied the structural equation model with Mplus to examine the CFA model and research hypothesis. Structural equation modeling was used to conduct the hypotheses test and model fitness through the statistical tool Mplus. Results imply that the data is suitable for conducting replication studies. To enlighten space tourism emergence studies, this data shows its importance for further research models [5].

**Specifications Table**

**Table:** 

Subject	Tourism, Leisure and Hospitality Management
Specific subject area	Space tourism is primarily about knowledge and practices of human space travel for recreational purposes. The area is different from earth Tourism because it involves the measurement scale of spacetime, which is the theoretical foundation of General Relativity.
Type of data	Raw data Table
How the data were acquired	Survey (the supplementary material is attached) The data was acquired through a survey with a questionnaire, which is designed through a literature review and adjusted by reliability and validity tests.
Data format	Raw Analyzed Filtered
Description of data collection	Data were collected by recruiting 361 respondents through snowball and convenient sampling targeted to samples interested in space tourism; 339 responses were adopted after the valid screening of missing data or data bias [4]. Targeted samples are potential customers who were investigated to collect data on a platform, Wenjuanxing, with a majority population database providing functions equivalent to Amazon Mechanical Turk [6]. We delivered the designed questionnaire to samples who are interested in space tourism. The reliability and validity of all constructs showed that the questionnaire was proper for measurement [7]. Data analysis applied the structural equation model with Mplus to examine the CFA model and research hypothesis. Structural equation modeling was used to conduct the hypotheses test and model fitness through the statistical tool Mplus. Results imply that the data is suitable for conducting replication studies.
Data source location	*· City/Town/Region:* Macau SAR *· Country:* Chinese Mainland
*Data accessibility*	*Repository name: Mendeley Data* *Direct URL to data:*https://data.mendeley.com/datasets/92bdw68ctw/4
*Related research article*	*Peng, K.*L.*, Hsu, C.H.C., Lin, P.M.C., & Su, M. (2022). Proposing spacetime scale for space tourism economics. Tourism Economics, 13,548,166,221,109,666.*https://doi.org/10.1177/13548166221109666

**Value of the data**

• The data is useful because it is applied to research models related to the constructs of social media, flow, and behavior intention. It could also expand to more constructs to conduct competitive statistical models of space tourism studies. The measurement scales have been tested with good reliability and validity that could be replicated or adjusted for longitudinal studies. The data has been conducted SEM test with a good model fit of a space tourism study that shows the value in data science.
• Most space tourism research is conceptual because data from the nascent industry is limited [2]. Thus this data is constrictive for conducting empirical studies to contribute to quantitative analysis in space tourism [3].
• Professors can also use the data to illustrate a course in statistical exploitation of survey data that focuses on structural equation modeling. The scientific and pedagogical orientations can be highlighted in teaching and learning. The varying responses could tell the various learning effects.
• The data could be applied to conduct replicated studies with competitive statistical models to compare the goodness of fit for observing the insights of space tourism studies. In addition, the data can also conduct cross-regions worldwide. The cross-region studies could statistically extend the research generalizability if the data could be collected more out of China. As argued above, further studies could be conducted to compare space tourism behavior intention with various regional policies while obtaining different datasets from other countries.
• The data is one of the empirical studies of the related research article, a published research note for a conceptual study of space tourism [1]. Only a few tourists have experienced real space trips, meaning consumer data with real experiences is rare. The data for investigating potential customers' behavioral intentions is a direction to conduct a space tourism study at this early stage.

## 1 Objective

The data is generated for an empirical study of the demand side study of space tourism. It is an extensive study for the related research article [1], which is a conceptual article with a different aim of economic measurement scale. Though, there have been a few tourists who have personal experience that can conduct empirical studies. We observe that many people are interested in space tourism, searching for and sharing related information on social media. Thus, the data were collected to study consumer behaviors from the casualties of social media, flow, and the behavioral intention of the potential tourists. This empirical study and the following research echo the related research article's leading perspective on space tourism.

## 2 Data Description

The raw data is measured from the questionnaire designed by the research team based on the literature review of the research constructs [8]. The questionnaire items are in Table 1, showing the variables' measurement scales. Q5∼Q7 variables are the second level of the social media construct with Cronbach α 0.934, which is categorized into 1) the entertaining characteristic, 2) the authenticity characteristic, and 3) the usefulness characteristic of social media content. Q8∼Q10, are the second level of the flow construct with Cronbach α 0.940, which measures 1) happiness, 2) concentration and 3) impressiveness of the flow experience. Q11 questions measure the attitude towards space tourism with Cronbach α 0.786. The descriptive analysis of questionnaire items is presented in Table 1 for reference. The rest of the questions are the demographical measure scales with descriptive analysis results, as indicated in Table 2. To show the normality of the constructs, and items, the skewness and kurtosis were presented in Table 3. The common method bias (CMB) was tested for carrying out SEM analysis. Table 4 shows that the model fit is poor without CMB through one-way confirmatory factor analysis. The questionnaire and raw data are provided on Mendeley data at https://data.mendeley.com/datasets/92bdw68ctw/4.

**Table 1:** The questionnaire and variable descriptive analysis.

Variables (Cronbach α)	Questions	Mean	St. Dev.
Q5	**The entertaining characteristic of social media content**		
(0.849)	a1. Social media content about space tourism relaxes me.	4.06	0.948
	a2. The content about space tourism on social media is interesting.	4.20	1.075
	a3. The content on social media about space tourism is entertaining.	3.67	1.097
Q6	**The authenticity characteristic of social media content.**		
(0.801)	b1. The message of space tourism on social media is real.	3.58	1.113
	b2. The information of space tourism on social media is accurate.	3.78	1.209
	b3. The information of space tourism on social media is reliable.	3.52	1.149
	b4. The information of space tourism on social media is trustworthy.	3.69	1.158
Q7	**The usefulness characteristic of social media content.**		
(0.840)	c1. The content of space tourism on social media advises me about space travel.	3.67	1.132
	c2. The content of space tourism on social media provides me with useful information.	4.24	1.016
	c3. The content of space tourism on social media helps me make travel decisions.	4.04	0.974
Q8	**Measuring the happiness of the flow experience.**		
(0.818)	d1. The content of space tourismon on social media gives me fun.	3.67	1.131
	d2. I enjoy the content of space tourism on social media.	3.47	1.094
	d3. The content of space tourism on social media makes me happy.	3.66	1.107
	d4. Overall, I'm willing to browse about space tourism content.	3.90	1.263
Q9	**Measuring the concentration of the flow experience.**		
(0.767)	g1. When browsing social media content about space tourism, I don't notice the passage of time.	4.21	1.012
	g2. When browsing social media content about space tourism, I don't notice what's going on around me.	3.42	1.086
	g3. When browsing social media content about space tourism, I am engrossed.	3.54	1.146
Q10	**Measuring the impressiveness of the flow experience.**		
(0.887)	l1. When browsing social media content about space tourism, it seems that the scenes (rockets, launchers, cosmos, spaceships) are real.	4.27	1.006
	l2. When browsing social media content about space tourism, it seems that the description of the sensation (weightlessness, impact) is real.	3.73	1.174
	l3. When browsing social media content about space tourism, I feel that I can touch the people and objects of the content.	4.17	1.040
	l4. When browsing social media content about space tourism, I feel that I am in a spaceship floating in space.	4.14	1.022
Q11	**The attitude towards space tourism.**		
(0.786)	m1. I would like to try space tourism if possible.	3.52	1.091
	m2. I would choose space tourism when I get a chance.	3.92	1.233
	m3. In general, I want to go for space tourism.	4.17	0.990

**Table 2:** Descriptive analysis of samples.

Variables	Questions	Frequency	Percentage
**Q12**	**Your gender**		
	Male;	166	49%
	Female	173	51%
**Q13**	**Your age**		
	18–25	132	39%
	26–35	121	36%
	36–45	74	22%
	46–55	4	1%
	56 or above	8	2%
**Q14**	**Education level**		
	Junior high or below	71	21%
	Senior high	73	22%
	Bachelor	146	43%
	Master or above	49	15%
**Q15**	**Occupation**		
	Private company staff	62	18%
	Soldier	18	5%
	Retirees	8	2%
	State unit personnel	47	14%
	Student	82	24%
	Government staff	57	17%
	Freelancer	65	19%
**Q16**	**Region**		
	Mainland China	317	94%
	Hong Kong, Macau, and Taiwan	22	6%
**Q17**	**Income level**		
	15,001and above	38	11%
	10,001∼15,000	66	19%
	5001∼10,000	101	30%
	2001∼5000	95	28%
	2000 and below	39	12%

**Table 3:** The skewness and kurtosis of constructs and items.

Construct	Item	Skewness	Kurtosis	Mean
Entertainment	A1	−1.529	2.563	4.06
	A2	−1.734	2.558	4.2
	A3	−0.803	0.194	3.67
Authenticity	B1	−0.434	−0.34	3.58
	B2	−0.799	−0.166	3.78
	B3	−0.354	−0.41	3.52
	B4	−0.669	−0.194	3.69
Usefulness	C1	−0.56	−0.312	3.67
	C2	−1.694	2.626	4.24
	C3	−1.637	2.925	4.04
Happiness	D1	−0.576	−0.292	3.67
	D2	−0.248	−0.323	3.47
	D3	−0.818	0.211	3.66
	D4	−0.965	−0.063	3.90
Concentration	G1	−1.688	2.71	4.21
	G2	−0.348	−0.0109	3.42
	G3	−0.371	−0.413	3.54
Impressiveness	L1	−1.639	2.262	4.27
	L2	−0.701	−0.218	3.73
	L3	−1.66	2.483	4.17
	L4	−1.624	2.466	4.14
Attitude	M1	−0.27	−0.408	3.52
	M2	−0.872	−0.27	3.92
	M3	−1.63	2.668	4.17

**Table 4:** The test of common method bias.

χ²	df	χ²/df	RMSEA	CFI	TLI	SRMR
297.35	39	7.624	0.25	0.75	0.63	0.158

## 3 Experimental Design, Materials and Methods

**Fig. 1:**
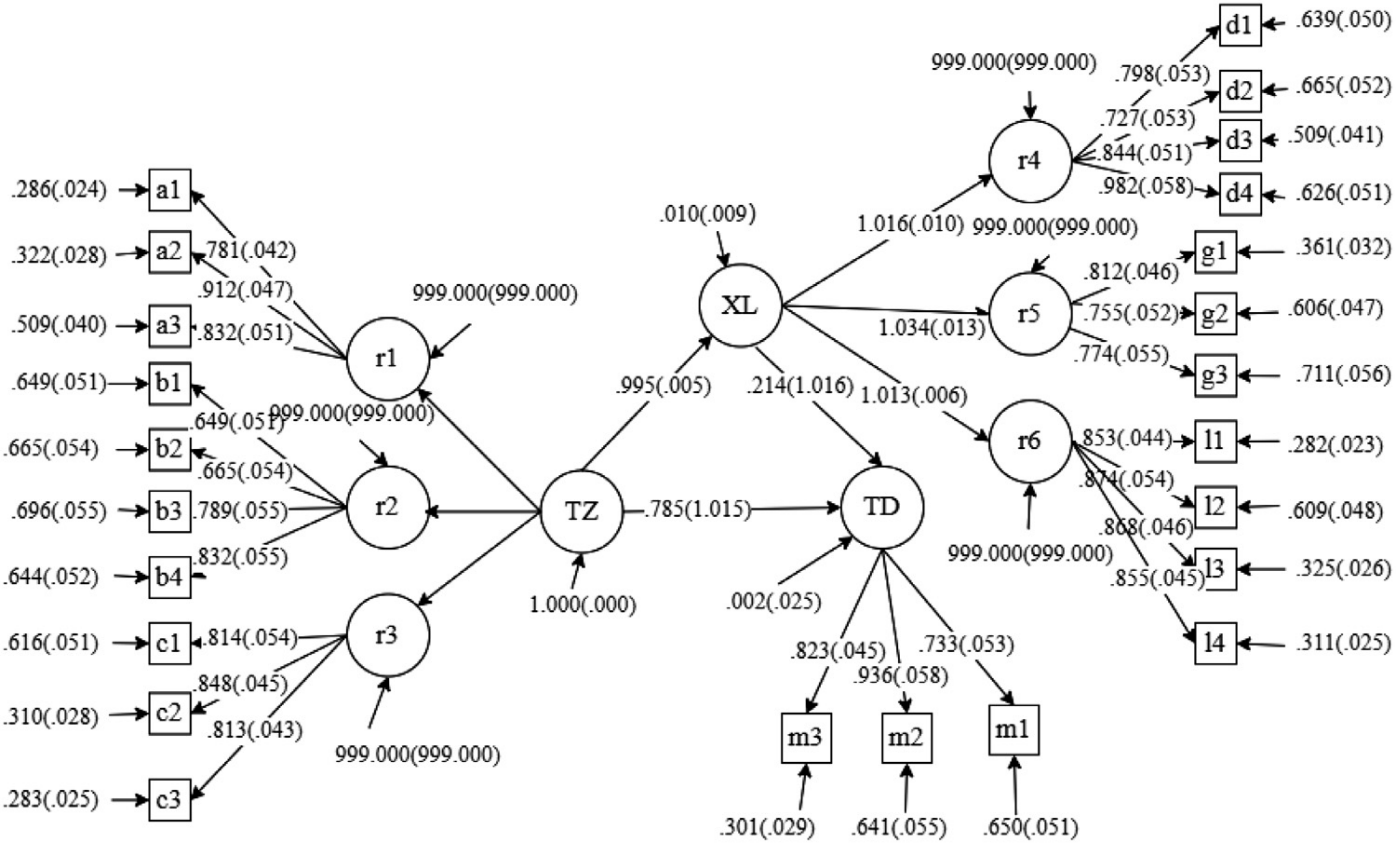
The data analyzed through SEM.

Table 5 shows the experimental design from the model construction to the analysis method. Based on the S-O-R model, the research design began with the research model and hypotheses construction, the causalities of social media, flow, and attitude. Then a questionnaire was developed with measurement scales of variables referred to and supported by the literature. The raw data were generated through a survey that measured participants' cognitions of the model constructs. The researchers collected a sample size of 361 valid responses. A snowball and convenient sampling of targeted groups were required on the survey platform Wenjuanxing with a majority population database providing functions equivalent to Amazon Mechanical Turk [6]. The data can be referred to as the population attribute from the sample file that might have a generalizability limit in the region item, as indicated in Table 2. The measurement scales meet essential reliability and validity requirements. The average variance extracted values are all above 0.5 to fit the validity criteria. Cronbach's alpha values ranged from 0.786 to 0.940, all above 0.7. Table 6 shows the validity criteria. Table 7 presents the discriminant validity test result through the AVE-correlation comparison. Each variable's AVE square root values are larger than the lower left triangle numbers, reflecting the constructs' discriminant validity [9]. We applied the SEM analysis method through the statistical tool Mplus to test research hypotheses corresponding to the research model. The data analyzed through SEM is indicated in Fig. 1.

**Table 5:** The experimental design process.

Process	Design	Criteria or Purpose
Step 1	Model and hypotheses	Literature support
Step 2	Measurement scales development	Reliability and validity tests
Step 3	Sampling strategy: Random sampling	Representative to the population
Step 4	Data collection: Wenjuanxing	Sample size
Step 5	Data curation	Valid data
Step 6	Data analysis methods: SEM	Hypotheses test and Model fitness
Step 7	Statistics tool: Mplus	Statistical flexibility

**Table 6:** The validity test.

Construct	Item	Estimate	S.E.	Est./S.E.	P-Value	SMC	CR	AVE
Entertainment	A1	0.781	0.042	18.508	0.000	0.390	0.880	0.711
	A2	0.912	0.047	19.357	0.000	0.168		
	A3	0.832	0.051	16.454	0.000	0.308		
Authenticity	B1	0.765	0.053	14.391	0.000	0.585	0.892	0.673
	B2	0.891	0.057	15.689	0.000	0.794		
	B3	0.789	0.055	14.337	0.000	0.623		
	B4	0.832	0.055	15.196	0.000	0.692		
Usefulness	C1	0.814	0.054	15.073	0.000	0.663	0.865	0.681
	C2	0.848	0.045	18.759	0.000	0.719		
	C3	0.813	0.043	18.833	0.000	0.661		
Happiness	D1	0.798	0.053	14.937	0.000	0.637	0.907	0.710
	D2	0.727	0.053	13.746	0.000	0.529		
	D3	0.844	0.051	16.495	0.000	0.712		
	D4	0.982	0.058	16.960	0.000	0.964		
Concentration	G1	0.812	0.046	17.629	0.000	0.659		
	G2	0.755	0.052	14.627	0.000	0.570	0.824	0.609
	G3	0.774	0.055	13.976	0.000	0.599		
Impressiveness	L1	0.853	0.044	19.420	0.000	0.728	0.921	0.744
	L2	0.874	0.054	16.051	0.000	0.764		
	L3	0.868	0.046	18.969	0.000	0.753		
	L4	0.855	0.045	19.014	0.000	0.731		
Attitude	M1	0.733	0.053	13.902	0.000	0.537	0.872	0.697
	M2	0.936	0.058	16.168	0.000	0.876		
	M3	0.823	0.045	18.380	0.000	0.677

**Table 7:** The discriminant validity test.

	Entertain-ment	Authen-ticity	Usefulness	Happiness	Concentra-tion	Impressive-ness	Attitude
**Entertainment**	**0.843**						
**Authenticity**	0.683	**0.820**					
**Usefulness**	0.662	0.637	**0.825**				
**Happiness**	0.539	0.617	0.720	**0.843**			
**Concentration**	0.506	0.615	0.708	0.764	**0.780**		
**Impressiveness**	0.577	0.635	0.631	0.692	0.643	**0.863**	
**Attitude**	0.620	0.566	0.684	0.745	0.730	0.675	**0.835**

*Note:* The numbers in bold diagonal indicate each variable's AVE square root value; The numbers in the lower left triangle area indicate the correlation coefficient between the variables.

## Ethics Statements

All authors comply with research ethics for the data. The study acquired ethics approval from the Human Research Ethics Committee for Non-Clinical Faculties of the City University of Macau. The data are anonymized adequately so that participants can not be identified, and informed consent was offered at the time of original data collection through the survey platform Wenjuanxing, whose data redistribution policies complied with the ethical requirement. The consent form does not need to obtain the subjects' signatures; such waiver is applicable because we use an internet survey anonymously.

## CRediT Author Statement

**Kang-Lin PENG**: Conceptualization, Methodology, Data curation, Formal analysis, Model construction, Writing- Original draft preparation, Supervision. **Xianglin CHAN**: Software, Data curation, Writing- Reviewing and Editing, Resources. **Chih-Hung WU**: Resources, Data curation, Validation.

## Declaration of Competing Interest

The authors declare that they have no known competing financial interests or personal relationships that could have appeared to influence the work reported in this paper.

## Data Availability

Data_Space tourism flow (Original data) (Mendeley Data)
